# *β*-Thalassemia: HiJAKing Ineffective Erythropoiesis and Iron Overload

**DOI:** 10.1155/2010/938640

**Published:** 2010-05-19

**Authors:** Luca Melchiori, Sara Gardenghi, Stefano Rivella

**Affiliations:** Weill Cornell Medical College, Department of Pediatrics, Division of Hematology-Oncology, 515E 71st street, S702, New York, NY 10021, USA

## Abstract

*β*-thalassemia encompasses a group of monogenic diseases that have in common defective synthesis of *β*-globin. The defects involved are extremely heterogeneous and give rise to a large phenotypic spectrum, with patients that are almost asymptomatic to cases in which regular blood transfusions are required to sustain life. As a result of the inefficient synthesis of *β*-globin, the patients suffer from chronic anemia due to a process called ineffective erythropoiesis (IE). The sequelae of IE lead to extramedullary hematopoiesis (EMH) with massive splenomegaly and dramatic iron overload, which in turn is responsible for many of the secondary pathologies observed in thalassemic patients. The processes are intimately linked such that an ideal therapeutic approach should address all of the complications. Although *β*-thalassemia is one of the first monogenic diseases to be described and represents a global health problem, only recently has the scientific community started to focus on the real molecular mechanisms that underlie this disease, opening new and exciting therapeutic perspectives for thalassemic patients worldwide.

## 1. Introduction

The biochemical signature of *β*-thalassemia is a reduced synthesis of the *β*-globin subunit of HbA (*α*2*β*2). Individuals inheriting two *β*-thalassemic alleles experience a profound deficit in *β*-chain production, and this impairment leads to excess production of *α*-globin. No compensatory regulatory mechanism exists where impaired synthesis of *β*-globin subunit leads to an excess production of the *α*-globin. Therefore, in *β*-thalassemia, the excess *α*-globin chains form tetramers that accumulate and precipitate in the erythroid progenitors, forming inclusion bodies that cause oxidative membrane damage within the red blood cells and immature developing erythroblasts in the bone marrow. This leads to premature death of many late erythroid progenitors in the bone marrow and spleen (Hoffman et al., Hematology, Basic Principles and Practice). The profound anemia that results from production of only a few hypochromic and microcytic red blood cells leads to a dramatic increase in erythropoietin (EPO) levels, that ultimately drive an uncontrolled expansion of additional early erythroid progenitors inducing massive EMH. These erythroid progenitors have an enhanced proliferative and survival capacity, but eventually they fail to differentiate, contributing to the process of IE [1]. The standard treatment for thalassemic patients is chronic blood transfusion to ameliorate the hemoglobin level. Chronic transfusion therapy is required to sustain life in patients with *β*-thalassemia major and often becomes necessary in those with *β*-thalassemia intermedia who develop splenomegaly. The main side effect of transfusion therapy is the dramatic iron overload. Secondary iron overload is indeed one of the major causes of morbidity in thalassemic patients. Excessive iron in the circulation leads to abnormal accumulation in organs such as liver, spleen and heart, leading ultimately to liver disease, cardiac dysfunction, arthropathy, gonadal insufficiency, and other endocrine disorders (Hoffman et al., Hematology, Basic Principles and Practice). *β*-thalassemia is also an iron loading anemia, meaning that thalassemic patients have a dramatic increase in iron absorption from the gut due to their increased erythropoietic rate. This increased iron absorption is mainly mediated by downregulation of the ironregulatory hormone hepcidin [2–9], and together with the iron influx from chronic transfusions contributes to the general setting of iron overload observed in thalassemic patients. Current treatment for iron overload includes administration of iron chelators like desferoxamine, deferasirox, and deferiprone [10]. Those agents decrease the iron burden in the liver and heart, significantly increasing the lifespan of thalassemic patients. Splenectomy is also often necessary to contain the iron burden when the transfusion requirement becomes excessive (*β*-thalassemia major) or the anemia worsens (*β*-thalassemia intermedia). Splenectomy, however, introduces a new set of problems. Splenectomized patients have an increased risk of infections, of developing thrombotic events, and of pulmonary hypertension, all life-threatening conditions [11–14]. These observations suggest a reconsideration of splenectomy in thalassemic patients. The use of agents able to reduce the spleen size thereby allowing patients to avoid splenectomy could be an efficient option in those who develop splenomegaly. All of the approaches described above target secondary pathologies that result from IE in *β*-thalassemia. Therefore, treatment of IE itself could ameliorate all secondary pathologies that stem from it.

## 2. Jak2 and Erythropoiesis

During the erythropoietic process, multipotent stem cells in the bone marrow divide and differentiate giving rise to approximately 2 million nonnucleated reticulocytes every second. This process is tightly regulated by an array of events that include cytokine signaling and cell-cell interactions, mainly in the context of erythroblastic islands, a specialized niche for the maturation of erythroid progenitors [15]. Since the pivotal role of erythrocytes is carrying oxygen to tissues, the erythropoietic process must be able to respond quickly and effectively to changes in tissue oxygen tension. This is accomplished mainly by erythropoietin (EPO). EPO serves as the master regulator of erythropoiesis [16]. It signals through the erythropoietin receptor (EPOR) and controls virtually all stages of erythroid differentiation, from committed common myeloid progenitors to the survival, proliferation, and maturation of more late-stage erythroid progenitors such as proerythroblasts and basophilic erythroblasts [17]. EPO exerts its effects by binding to EPOR, thereby activating the cytoplasmatic kinase Jak2. Activation of Jak2 involves auto and crossphosphorylation events that ultimately lead to activation of the signal transductor and activator of transcription Stat5 a and b and parallel signaling pathways [18]. Once activated, Stat5 migrates to the nucleus and activates genes crucial for proliferation, differentiation, and survival of erythroid progenitors. The crucial importance of the EPO-EPOR-JAK2-STAT5 axis has been demonstrated by knock out studies in mice that showed how lack of each one of these four molecules results in a lethal phenotype (death due to severe anemia) during fetal development [19]. The binding of EPO to EPOR leads to activation of another important signaling pathway during erythropoiesis: the phosphoinositol-3-kinase (PI3K)—AKT pathway [20]. Studies in an apoptosis-resistant erythroid cell line indicate that activation of PI3K is necessary but not sufficient to protect against apoptosis [21]. Moreover, mice that do not express the p85 *α* subunit of PI3K have dramatically reduced erythropoiesis with reductions in the numbers of CFU-E and BFU-E progenitors [22]. AKT, which is the activated downstream of PI3K, transduces signals that are necessary for the differentiation of erythroid progenitors [23] and is important in regulation of the activity of the FOXO3 transcription factor. The activity of FoxO3 is pivotal in the regulation of oxidative stress during erythropoiesis, as knockout mice exhibit a greater susceptibility to ROS-induced oxidative stress [24]. The absence of FoxO3 was associated with reduction in erythrocyte lifespan as well as an enhanced mitotic arrest in intermediate erythroid progenitor cells, resulting in a decreased rate of erythroid maturation. FoxO3-null erythrocytes also showed decreased expression of ROS scavenging enzymes and evidence of oxidative damage. Jak2 is a member of the Jak tyrosine kinase family, and mediates the action of many other cytokines besides EPO. Some examples include growth hormone, prolactin, TPO, GM-CSF, interleukin 3, and interleukin 5. What makes the association between EPO and Jak2 unique is the fact that Jak2 is the only kinase that is associated with EPOR and therefore the only signal transductor of EPO. This important task is accomplished by a complex 3D structure that involves kinase, pseudokinase, and regulatory domains. The 3D structure allows for a finely tuned regulation of its activity, from its association with EPOR to its phosphorylation and activation [25]. When regulation fails, the consequences are deleterious. In 2005, five different research teams [26–30] described an activating mutation (V617F) in the pseudokinase domain of Jak2 that is associated with ≥90% of cases of polycythemia vera and ~50% of cases of essential thrombocythemia and chronic idiopathic myelofibrosis. In all of these studies, it was clear how hyperactivation of Jak2 contributes to a massive increase in the erythropoietic activity and a huge expansion of the erythron with related splenomegaly, a phenotype that is interestingly similar to what is observed in *β*-thalassemia.

### 2.1. Jak2, Ineffective Erythropoiesis and Splenomegaly in *β*-Thalassemia

**Figure 1:**
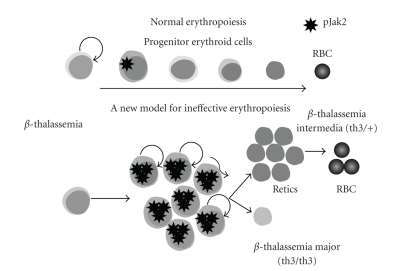
In normal erythropoiesis, physiological levels of EPO induce the phosphorylation of Jak2 in normal erythroid progenitors and sustain the differentiation to mature RBC (top of the figure). In *β*-thalassemia, the high levels of EPO induce an uncontrolled proliferation of erythroid precursors, with a higher number of cells associated with the phosphorylated form of Jak2. In *β*-thalassemia intermedia, where a certain amount of *β*-globin is still synthesized, there is a high production of reticulocytes that eventually mature in RBC. In *β*-thalassemia major, where there is a complete lack of *β*-globin, the orythroid progenitors continue to proliferate and fail to mature into reticulocytes and RBC or undergo apoptosis.

In *β*-thalassemia, the erythropoietic process is markedly altered and is referred to as ineffective erythropoiesis (IE). According to the traditional model, the lack or reduced synthesis of *β*-globin during IE induces the formation of *α*-globin aggregates in erythroid progenitors. These aggregates precipitate and adhere to the membrane causing cellular damage, massive apoptosis of erythroid progenitors in the bone marrow, and only limited production of red blood cells which are abnormal. These abnormal/damaged RBC are readily captured by the reticuloendothelial system in the spleen and contribute to the splenomegaly observed in thalassemic patients. The hypoxia arising from the lack of production of normal RBC induces a dramatic increase in the levels of EPO, which in turn induces a bone marrow hyperplasia and bone deformities. This traditional view has mainly focused on the apoptotic aspect of IE [31–33], which is an important but not the only aspect of this process. Recent studies using both mouse models of *β*-thalassemia and specimens from thalassemic patients showed that, together with apoptosis, a significant number of erythroid progenitors undergo increased proliferation and decreased differentiation in the spleen [1]. In this model, high levels of EPO act as the driving force for the survival and proliferation of erythroid progenitors, albeit they fail to efficiently differentiate giving rise to only a few RBCs. This process has been shown to be associated with the phosphorylated form of Jak2, leading to a higher number of proliferating thalassemic erythroid progenitors compared to normal conditions, in a sort of “physiological” gain of function (Figure 1). The persistent phosphorylation of Jak2 as a consequence of high EPO levels induces a massive extramedullary hematopoiesis (EMH), with early erythroid progenitors colonizing then proliferating mainly in the spleen and liver. In this scenario, the spleen becomes a secondary erythropoietic niche, with its enlargement (splenomegaly) being due mainly to the colonization and proliferation of erythroid progenitors from the bone marrow. In our study, we showed that erythroid progenitors derived from the blood of thalassemic patients express higher levels of cell cyclerelated mRNAs such as Jak2, Ki67, Cyc A, BclXL, and EpoR, and that there are a considerable number of erythroid progenitors in the spleen of thalassemic patients that are actively proliferating. Moreover, recent studies have shown that, at least in the mouse, there are erythroid progenitors that develop in the spleen, with different capacities for proliferation and differentiation than the ones found in the bone marrow. These erythroid progenitors display a higher sensitivity to EPO and are selectively responsive to BMP4. They show an improved capacity to respond to acute anemia and a higher differentiation rate compared to their counterparts in the bone marrow [34]. Another important study shows how the transcription factor ID1 is directly upregulated by the Jak2-Stat5 pathway in erythroid cells [35]. Since high levels of ID1 have been found to inhibit cell differentiation, its up-regulation due to a sustained activation of Jak2 by EPO in *β*-thalassemia could explain the less mature erythroid progenitors observed. These findings shed light on the mechanisms that underlie splenomegaly and raise the intriguing question of whether there is also a basal physiological level of erythropoietic activity in the human spleen that is dramatically increased in stress conditions such as IE. Although these observations have been demonstrated in mouse models, further evaluation is necessary in humans, especially considering the differences observed in the erythropoiesis in the spleen comparing these two species. Other mechanisms could be involved in the onset of splenomegaly, like hepatic and portal vein obstruction, cirrhosis or congestive heart failure that often appear in thalassemic patients. All these conditions lead to an increased blood flow to the spleen that in turn could be responsible for the enlargement observed.

### 2.2. Possible Role of Jak2 Inhibitors in *β*-Thalassemia

The discovery of Jak2 as an important mediator of IE and splenomegaly in *β*-thalassemia suggests that the use of small organic molecules to inhibit Jak2 could be beneficial in reducing IE and splenomegaly. The idea of treating an erythropoietic disorder with an agent that limits erythropoiesis appears counterintuitive. However, the rationale for its use is related to the fact that IE in *β*-thalassemia resembles a leukemic blast expansion, with immature erythroid progenitors that proliferate abnormally, fail to differentiate, and invade other organs to compromise their function [1]. It is important to state that this is still a model that needs further confirmation, since mouse models of *β*-thalassemia and leukemia have not been compared yet. Ideally, use of a Jak2 inhibitor would target only the rapidly proliferating erythroid progenitors in the spleen (CD71+ Ter119+ early erythroid progenitors), blocking their expansion and therefore allowing shrinkage of this organ by decreasing the presence of red pulp. This in turn would contribute to an improvement of the spleen architecture and a reduction of RBC sequestration, enhancing their lifespan. Jak2 inhibitors are indeed able to induce a dramatic decrease in spleen size [1] in thalassemic animals with a limited effect on anemia. The drug would need to be carefully titrated in order to achieve an optimal plasma concentration that would allow the inhibition of excessive proliferation or early progenitors without blocking erythropoiesis, as it would be expected by completely inhibiting Jak2 [19]. This titration process would also limit the potential for off-target effects on the immune system by a Jak2 inhibitor, effects that however have not been found relevant in other mouse models [36]. However, it is important to point out that *β*-thalassemia major and *β*-thalassemia intermedia patients who develop splenomegaly require regular blood transfusions and often undergo splenectomy. It is appealing to speculate that *β*-thalassemia intermedia patients affected by splenomegaly could be treated temporarily with Jak2 inhibitors so as to reduce the spleen size and, in the presence of blood transfusions, to prevent further anemia. This suggests that even patients affected by *β*-thalassemia major, who develop splenomegaly and EMH, may benefit from administration of Jak2 inhibitors (Melchiori et al., in preparation). In these settings, the use of Jak2 inhibitors would be expected to limit or reduce splenomegaly, thereby preventing or delaying the need for splenectomy and indirectly improving the management of anemia and iron overload by reducing the rate of blood transfusions. In conclusion, the use of Jak2 inhibitors for *β*-thalassemia might be desirable, but it would require a careful optimization noting the potential for off-target immune suppression, as well as the anemia that would be expected from continuous Jak2 inhibition.

### 2.3. Iron Overload as a Consequence of Ineffective Erythropoiesis

Erythropoiesis and iron metabolism are closely linked processes. The large majority of iron in our body is utilized by the erythropoietic system to generate functionally active hemoglobin molecules, which are harbored in the RBC. It is estimated that 1800 mg of iron in our body are present in RBCs, 300 mg in the bone marrow, and 600 mg in the reticuloendothelial macrophages of the spleen [37], accounting for more than 60% of total body iron. This massive utilization of iron by the erythropoietic system requires the presence of finely tuned regulatory systems that allow storage, mobilization, and traffic of iron while preventing toxicity due to highly reactive iron ions. It is also important to remember that our body lacks an efficient means of iron excretion. Therefore, its regulation occurs primarily at the site of absorption in duodenal enterocytes. Other important regulatory sites are the liver, where large quantities of iron can be stored in hepatocytes and Kuppfer cells, and the spleen, where macrophages recycle iron from senescent RBCs. All three compartments are of pivotal importance for erythropoiesis since they control the bioavailability of iron to erythroid progenitors, and all of them respond to what has been found to be the master regulator of iron metabolism, the peptide hormone hepcidin. Hepcidin [38, 39] is synthesized mainly in the liver, and when released into the blood stream, binds to ferroportin, the major cellular iron exporter expressed at high levels on the surface of duodenal enterocytes, liver hepatocytes, and Kuppfer cells, and splenic macrophages [40–43]. The binding of hepcidin to ferroportin induces its internalization and destruction in the cellular proteasome [44, 45]. Therefore, the main function of hepcidin is to reduce iron absorption from enterocytes, and to limit iron export and trafficking from hepatocytes and splenic macrophages. The action of hepcidin is critical in conditions of iron overload, when too much iron is potentially bioavailable and a drastic reduction in its absorption and mobilization is required. Hepcidin expression is strictly regulated, with multiple pathways that respond to iron storage (storage regulator) [46], hypoxia (hypoxia regulator) [47, 48], inflammation (inflammatory regulator) [49–52], and erythropoiesis (erythroid regulator) [51, 53, 54]. All these systems interact with one another resulting in a very complex and finely tuned regulation of hepcidin expression. Since the intimate relationship between hepcidin and erythropoiesis exists, it is predictable that pathologies that involve alteration in the erythropoietic rate, also involve alterations of iron metabolism and hepcidin expression. In *β*-thalassemia, the process of IE is accompanied by a massive iron overload, due to an increased rate of iron absorption by the gastrointestinal (GI) tract and to frequent blood transfusions. In this setting, two major systems would contribute to hepcidin expression, the “stores regulator” and the “erythroid regulator”. Due to the high levels of total body iron, the store regulator should act by increasing hepcidin expression thereby avoiding further iron absorption. On the other hand, the erythroid regulator would decrease hepcidin expression in an attempt to compensate for anemia due to IE. Our recent study in mouse models of *β*-thalassemia intermedia (th3/+) and major (th3/th3) showed that the erythroid regulator does dictate the pattern of iron absorption and distribution relative to the degree of IE (Gardenghi et al., [4]). In th3/+ mice, iron overload and the degree of IE gradually become more severe as the animals age. Gardenghi and colleagues demonstrated how dysregulation of iron absorption in young th3/+ mice is due mainly to a dramatic decrease of hepcidin expression in the liver. However, as iron overload progressively increases in older animals, hepcidin is upregulated, while ferroportin expression is increased in the GI tract in order to maintain high levels of iron absorption to compensate for the anemia in IE. In th3/th3 mice, where IE is more pronounced, the increased iron absorbed was not found in hematopoietic organs such as the spleen and bone marrow, but rather in the liver and other nonhematopoietic organs [4]. This suggests that that iron is not utilized by erythroid progenitors, as would be expected according to the model of IE described above where erythroid progenitors display an increased proliferative activity but a decreased differentiation rate, resulting in a limited synthesis of hemoglobin and therefore limited iron uptake. Instead, in th3/+ animals where the degree of IE is lower and there is a substantial effective erythropoiesis, an increased iron content is found in the spleen and Kupffer cells of the liver [4]. This leads to the conclusion that the erythroid regulator overrides the store regulator in th3/th3 mice, resulting in low levels of hepcidin expression and further increasing the iron concentration in the liver. In contrast, in states of relatively mild anemia, iron absorption would be lower and the erythroid organs, spleen and bone marrow, would utilize part of the absorbed iron, as is observed in th3/+ animals. The idea that not all the iron absorbed in *β*-thalassemia is utilized for erythropoiesis has been confirmed by our recent data (Gardenghi et al., submitted) in th3/+ animals kept on a low iron diet. These animals show a lower iron content compared to counterparts fed a regular diet, but do not display a decrease in hemoglobin levels, suggesting again that an excessive amount of iron is absorbed in *β*-thalassemia but is not utilized for the erythropoietic process.

### 2.4. Control of Hepcidin Expression by the Erythroid Regulator

New research data hints at the mechanisms by which the erythroid compartment controls hepcidin expression. In *β*-thalassemia, several factors have been identified and studied as candidate hepcidin regulatory proteins including growth differentiation factor 15 (GDF15) [55], and human twisted gastrulation factor (TWSG1) [56]. Both of these two factors are members of the TGFbeta superfamily, which controls proliferation, differentiation, and apoptosis in numerous cells, and are secreted by erythroid precursors. TWSG1 gene expression occurs early during erythroblast maturation contrasting with the more sustained increase in GDF15 expression in more mature hemoglobinized erythroblasts. GDF15, which is elevated in the sera of patients with *β*-thalassemia, has the ability to down regulate the expression of hepcidin in vitro, although the mechanism is still unclear. In fact sera from these patients also suppressed hepcidin expression, albeit to a lesser degree, after immunoprecipitation of GDF15 [55]. Therefore, GDF15 may play a role in hepcidin regulation when erythroid precursors undergo cell death as occurs in the IE observed in *β*-thalassemia and refractory anemia with ring sideroblasts (RARS) [57]. TWSG1 has been shown to inhibit the upregulation of hepcidin by bone morphogenic proteins 2 and 4 (BMP2, BMP4), mediated by Smad phosphorylation, in human hepatocytes but was not BMP-mediated in murine hepatocytes [56]. Tanno and colleagues proposed that TWSG1 might act with GDF15 to dysregulate iron homeostasis in *β*-thalassemia. These represent new and exciting findings that can lead to novel discoveries and clinical applications. These factors will likely result in additional studies leading to the potential characterization of novel pathways controlling hepcidin production, have the potential to be utilized as prognostic markers, and lead to novel clinically applicable therapy in the future.

### 2.5. New Therapeutic Approaches to Limit Iron Absorption

The strong feedback of increased erythropoietic rate which suppresses hepcidin raises an important question about whether agents that limit IE, such as Jak2 inhibitors, could act indirectly as inducers of hepcidin expression. Our recent data on th3/+ and transfused th3/th3 mice treated with a Jak2 inhibitor suggest that this might be the case. Animals treated with the drug showed increased levels of hepcidin in the liver compared to animals treated with a placebo. More importantly, the levels of hepcidin negatively correlated with the spleen weight in these animals, suggesting a strong connection between spleen weight, erythropoietic activity, and hepcidin expression in *β*-thalassemia. Jak2 inhibitors could have a more direct effect on iron metabolism. This is suggested by a recent study that shows how expression of transferrin receptor 1 (TfR1) on erythroid cells depends on Jak2-Stat5 signaling [58]. Perhaps the use of Jak2 inhibitors could reduce the expression of TfR1 on erythroid cells, limiting iron uptake and potentially reducing the toxicity induced by free iron ions. In this scenario, the therapeutic treatment of *β*-thalassemia patients with Jak2 inhibitors could be useful as it would target the two major complications of this pathology, IE with its related splenomegaly and the massive iron overload. Therefore, not only the erythroid cells would benefit from the lower iron load, but also liver parenchimal cells and cells in other tissues (i.e., heart) that are damaged in iron overload conditions. Another therapeutic approach to decrease iron overload might be to increase the expression of hepcidin in thalassemic patients. Our recent study (Gardenghi et al., submitted) showed that increasing the hepcidin levels in th3/+ mice both by administration of exogenous synthetic hepcidin and by overexpressing hepcidin reduces organ iron overload resulting in a marked beneficial effect on hepatosplenomegaly and erythropoiesis. This reveals a potential role for hepcidin or hepcidin agonists in the treatment of abnormal iron absorption in *β*-thalassemia and other related disorders.

## 3. Summary

**Figure 2:**
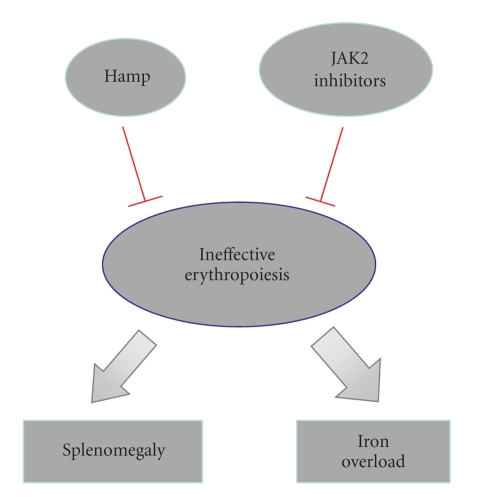
Different approaches to target IE in *β*-thalassemia. The administration of hepcidin would allow to decrease the iron overload in organs such the liver and heart and to redistribute the iron to hematopoietic organs, allowing a more efficient erythropoiesis and therefore a decrease in splenomegaly. The administration of Jak2 inhibitors would induce a decrease in the inefficient erythropoietic rate, therefore decreasing spleen size. The reduced erythropoiesis would have as indirect effect the increase in serum hepcidin, that in turn would decrease the iron absorption from the gut and the amelioration of iron overload.

Recent studies shed new light on the molecular mechanisms that underlie IE in *β*-thalassemia. These findings suggest that Jak2 plays a role in the onset of IE and splenomegaly, and show how the use of Jak2 inhibitors to limit these processes could open exciting new therapeutic options for thalassemic patients. The large body of work on hepcidin is starting to reveal evidence of what has long been held to be true, the existence of an “erythroid regulator”. The discovery of erythroid factors that regulate hepcidin production suggests possible new therapeutic targets to decrease iron overload. Furthermore, the evidence that exogenous hepcidin administration can ameliorate organ iron load without affecting anemia opens a new therapeutic field for hepcidin agonists/mimetics involving the treatment of different kinds of hemochromatosis (Figure 2).
